# Supplementary material for: Feasibility Study for the Development of a Low-Cost, Compact, and Fast Sensor for the Detection and Classification of Microplastics in the Marine Environment
Source: Sensors (Basel). 2023 Apr 19;23(8):4097. doi: 10.3390/s23084097 (PMC10143223; doi:10.3390/s23084097)
Supplement: Supplementary file 1 [file sensors-23-04097-s001.zip › sensors-2316305-supplementary.pdf]

Supplementary Material for

Feasibility study for the development of a low-cost, compact, and fast sensor for detection and classification of microplastics in marine environment

Bruno Cocciaro[1], Silvia Merlino[2*], Marco Bianucci[2], Claudio Casani[2,3], and Vincenzo Palleschi[1]

1 Consiglio Nazionale delle Ricerche—Istituto di Chimica dei Composti Organo-Metallici (CNR-ICCOM), U. O. S. di Pisa, Area della Ricerca del CNR, Via G. Moruzzi, 1, 56124 Pisa, Italy
2 Consiglio Nazionale delle Ricerche—Istituto di Scienze Marine (CNR-ISMAR), U. O. S. di Pozzuolo di Lerici, c/o Forte Santa Teresa—Loc. Pozzuolo di Lerici, 19032 Lerici, Italy,
3 Dipartimento di Biologia, Universitò di Pisa, Via L. Ghini, Italy,
* Correspondence: silvia.merlino@sp.ismar.cnr.it

In Figure S1 the logical scheme for the classification of microplastics with the tree photodiodes sensor.

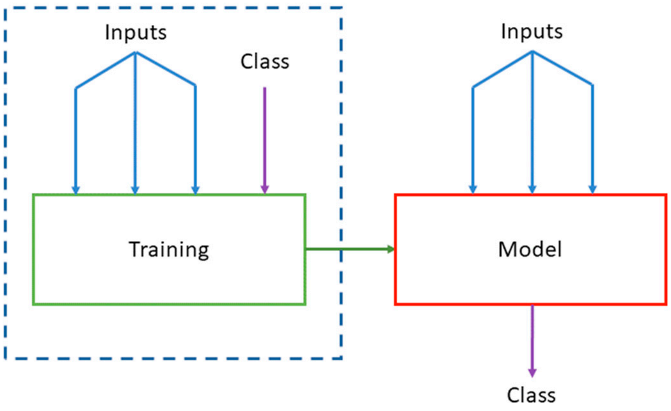

Figure S1. Logical scheme of the classification of the microplastics. A model linking the three inputs of the photodiodes to the corresponding class (PE or PP) is built after a proper training on objects whose classification is known. The classification of unknown samples is done applying the model to the corresponding signals of the photodiodes.

Below is the Matlab code used to recreate the classification model trained in the Classification Learner application. The code is self-explained in the commented preamble.

```
function [trainedClassifier, validationAccuracy] = trainClassifier(trainingData,
responseData)
% [trainedClassifier, validationAccuracy] = trainClassifier(trainingData,
% responseData)
% Returns a trained classifier and its accuracy. This code recreates the
% classification model trained in Classification Learner app. Use the
% generated code to automate training the same model with new data, or to
% learn how to programmatically train models.
%
```

```
%  Input:
%      trainingData: A matrix with the same number of rows and data type as
%       the matrix imported into the app.
%
%      responseData: A vector with the same data type as the vector
%       imported into the app. The length of responseData and the number of
%       columns of trainingData must be equal.
%
%  Output:
%      trainedClassifier: A struct containing the trained classifier. The
%       struct contains various fields with information about the trained
%       classifier.
%
%      trainedClassifier.predictFcn: A function to make predictions on new
%       data.
%
%      validationAccuracy: A double containing the accuracy as a
%       percentage. In the app, the Models pane displays this overall
%       accuracy score for each model.
%
% Use the code to train the model with new data. To retrain your
% classifier, call the function from the command line with your original
% data or new data as the input arguments trainingData and responseData.
%
% For example, to retrain a classifier trained with the original data set T
% and response Y, enter:
%   [trainedClassifier, validationAccuracy] = trainClassifier(T, Y)
%
% To make predictions with the returned 'trainedClassifier' on new data T2,
% use
%   yfit = trainedClassifier.predictFcn(T2)
%
% T2 must be a matrix containing only the predictor rows used for training.
% For details, enter:
%   trainedClassifier.HowToPredict

% Auto-generated by MATLAB on 10-Dec-2022 14:20:45


% Extract predictors and response
% This code processes the data into the right shape for training the
% model.
% Convert input to table
inputTable = array2table(trainingData', 'VariableNames', {'row_1', 'row_2', 'row_3'});

predictorNames = {'row_1', 'row_2', 'row_3'};
predictors = inputTable(:, predictorNames);
response = responseData;
isCategoricalPredictor = [false, false, false];

% Train a classifier
% This code specifies all the classifier options and trains the classifier.
classificationDiscriminant = fitcdiscr(...
    predictors, ...
    response, ...
    'DiscrimType', 'linear', ...
    'Gamma', 0, ...
    'FillCoeffs', 'off', ...
    'ClassNames', [1; 2]);

% Create the result struct with predict function
```

```
predictorExtractionFcn = @(x) array2table(x', 'VariableNames', predictorNames);
discriminantPredictFcn = @(x) predict(classificationDiscriminant, x);
trainedClassifier.predictFcn = @(x) discriminantPredictFcn(predictorExtractionFcn(x));

% Add additional fields to the result struct
trainedClassifier.ClassificationDiscriminant = classificationDiscriminant;
trainedClassifier.About = 'This struct is a trained model exported from Classification
Learner R2022a.';
trainedClassifier.HowToPredict = sprintf('To make predictions on a new predictor row
matrix, X, use: \n  yfit = c.predictFcn(X) \nreplacing ''c'' with the name of the
variable that is this struct, e.g. ''trainedModel''. \n \nX must contain exactly 3 rows
because this model was trained using 3 predictors. \nX must contain only predictor rows
in exactly the same order and format as your training \ndata. Do not include the
response row or any rows you did not import into the app. \n \nFor more information,
see <a href="matlab:helpview(fullfile(docroot, ''stats'', ''stats.map''),
''appclassification_exportmodeltoworkspace'')">How to predict using an exported
model</a>.');

% Extract predictors and response
% This code processes the data into the right shape for training the
% model.
% Convert input to table
inputTable = array2table(trainingData', 'VariableNames', {'row_1', 'row_2', 'row_3'});

predictorNames = {'row_1', 'row_2', 'row_3'};
predictors = inputTable(:, predictorNames);
response = responseData;
isCategoricalPredictor = [false, false, false];

% Perform cross-validation
partitionedModel = crossval(trainedClassifier.ClassificationDiscriminant, 'KFold', 5);

% Compute validation predictions
[validationPredictions, validationScores] = kfoldPredict(partitionedModel);

% Compute validation accuracy
validationAccuracy = 1 - kfoldLoss(partitionedModel, 'LossFun', 'ClassifError');
```
